# Phenotypic data on seedling traits of hexaploid spring wheat panel evaluated under heat stress

**DOI:** 10.1016/j.dib.2025.112069

**Published:** 2025-09-16

**Authors:** Santosh Gudi, Jatinder Singh, Harsimardeep Gill, Sunish Sehgal, Justin D. Faris, Upinder Gill, Rajeev Gupta

**Affiliations:** a Department of Plant Pathology, North Dakota State University, Fargo, ND, USA; b Department of Agronomy, Horticulture, and Plant Science, South Dakota State University, Brookings, SD, USA; c Edward T. Schafer Agricultural Research Center, USDA-ARS, Fargo, ND, USA

**Keywords:** Abiotic stress, BLUE, Heat stress, Wheat, Pearson’s correlation coefficient

## Abstract

Heat stress is the major abiotic stress affecting wheat at various developmental stages including seedling and reproductive stage. Heat stress at early developmental stages affects the seed germination and seedling establishment, thereby reduces grain yield per unit area. To overcome the negative impact of heat stress, it is crucial to identify the source of heat tolerant germplasm lines and also introduce them into breeding program. In this study, we evaluated 216 global diversity panel of hexaploid spring accessions comprising landraces and cultivars under non-heat stress (23 °C) and heat stress (36 °C) treatments. Phenotypic data was collected after 13 days of heat stress on various seedling traits, including coleoptile length (CL; cm), shoot length (SL; cm), root length (RL; cm), tiller number (TN), shoot fresh weight (SFW; mg), and root fresh weight (RFW; mg). Heat stress negatively affected all the seedling traits with maximum effect on RL (85.6 % reduction) and minimum effect on CL (15.44 %). However, the RN was increased by 20 % under heat stress. It was also noticed that the effect of heat stress was more on root traits (such as RL and RFW) as compared to shoot traits (such as SL and SFW). This suggests that compared to roots, shoots may have adaptive mechanisms such as transpiration cooling via stomatal regulation, to alleviate the negative impacts of heat stress. Moreover, the raw phenotypic data was subjected to mixed linear analysis to derive best linear unbiased estimates (BLUEs). BLUE values were further used to assess the intrinsic relationship among the seedling traits under non-heat stress (23 °C) and heat stress (36 °C) treatments. The dataset presented in this study serves a valuable source for identifying extremely tolerant lines for heat stress, which can be utilized in breeding program to develop heat resilient, high-yielding wheat cultivars. Moreover, this dataset helps in identifying potential genomic regions associated with improved heat stress tolerance, which can be incorporate in marker-assisted breeding of heat tolerant wheat varieties.

Specifications Table

**Table:** 

Subject	Biology
Specific subject area	Abiotic stress; Plant breeding
Type of data	*Tables and Figures*
Data collection	Seedlings were allowed to grow under non-heat stress (23 °C) and heat stress (36 °C) conditions up to 13 days after sowing. Plants were harvested on the 13th day and the roots were cleaned using running tap water. Coleoptile length was measured from scutellar node to the tip of coleoptile in centimetre (cm). SL was measured from scutellar node to the tip of topmost leaf, whereas the RL was measured from scutellar node to the tip of primary root in centimetre (cm). RN was determined by counting the main roots emerging from the radicle for each plant. Roots and shoots were separated by cutting plants at the scutellar node and the data on SFW and RFW measured in milligrams (mg).
Data source location	Heat stress screening and data collection was done in the growth chambers at Edward T. Schafer Agricultural Research Center, Fargo, ND, USA (46.892593 N and −96.807467 W).
Data accessibility	Repository name: Mendeley Data Data identification number: 10.17632/xdwfdr75xd.1 Direct URL to data: https://data.mendeley.com/datasets/xdwfdr75xd/1
Related research article	S. Gudi, J. Singh, H. S. Gill, S. K. Sehgal, J. D. Faris, U. Gill, R. Gupta (2025) Understanding the genetic basis of heat stress tolerance in wheat (*Triticum aestivum* L.) through genome-wide association studies. *The Plant Genome.* 18, e70071. https://doi.org/10.1002/tpg2.70071.

## 1 Value of the Data

• This dataset presents a protocol for high-throughput screening of global diversity panel including several germplasm lines in short period of time.
• The dataset presented in this study signifies the impact of heat stress on various seedling traits.
• This dataset identified contrasting genotypes with high-level of tolerance and susceptibility to heat stress, which can be utilized in developing bi-parental mapping populations.
• Heat tolerant germplasm lines identified from this dataset can be incorporate in breeding program to develop heat-resilient wheat cultivars.
• This dataset can be utilized in identifying potential genomic regions associated improved heat stress tolerance through genome-wide association study (GWAS) or through quantitative trait loci (QTL) mapping.

## 2 Background

Wheat is a staple food for >30 percent of the world population, which supplies ∼20 percent of calories and protein in the human diet [1]. In recent years, wheat productivity is reduced by fluctuating environmental conditions, such as increasing global temperature and unpredictable rainfall, thereby affecting global food security [2,3]. Major constraints for wheat productivity include, heat stress [4,5], drought stress [6], salt stress [7], lodging [8], nutrient deficiency [9], metal ion toxicity [10] etc. Heat stress is the major abiotic stress affecting wheat at various developmental stages, including early seedling stage and reproductive stages such as anthesis and grain filling. Heat stress during early developmental stages reduces the seed germination and seedling establishment, thereby significantly reduces grain yield and quality [6,11]. Heat stress alters cell integrity and membrane permeability by rupturing cell membrane and by inactivating the key enzymes responsible for plants growth and development. This results in the reduced photosynthetic assimilation, shoot-root biomass accumulation, and tiller number per plant [12]. Heat stress also affects cellular metabolism by increasing the reactive oxygen species (ROS), which results in stunted growth [13]. To overcome the negative effects of heat stress, it is necessary to identify the heat tolerant genotypes and introduce the potential genomic regions in cultivar background to develop heat resilient wheat cultivars. However, the quantitative nature of heat stress tolerance makes screening global diversity panel very difficult within short period of time. This problem can be resolved by phenotypic screening at early developmental stages, which helps in high-throughput evaluation of a large number of germplasm lines in short time. Moreover, seedling evaluation accelerates the breeding process by allowing early selection of promising genotypes. Therefore, in this study, we evaluated 216 globally diverse hexaploid spring wheat accessions including landraces and cultivars under non-heat stress (23 °C) and heat stress (36 °C) treatments at seedling stage. Observations were made on various seedling traits. This dataset helps in identifying extremely heat tolerant lines, which can be introduced in breeding program to develop heat resilient wheat cultivars. Moreover, this dataset can be utilized to identify candidate genomic regions responsible for improved heat stress tolerance in wheat.

## 3 Data Description

**Fig. 1:**
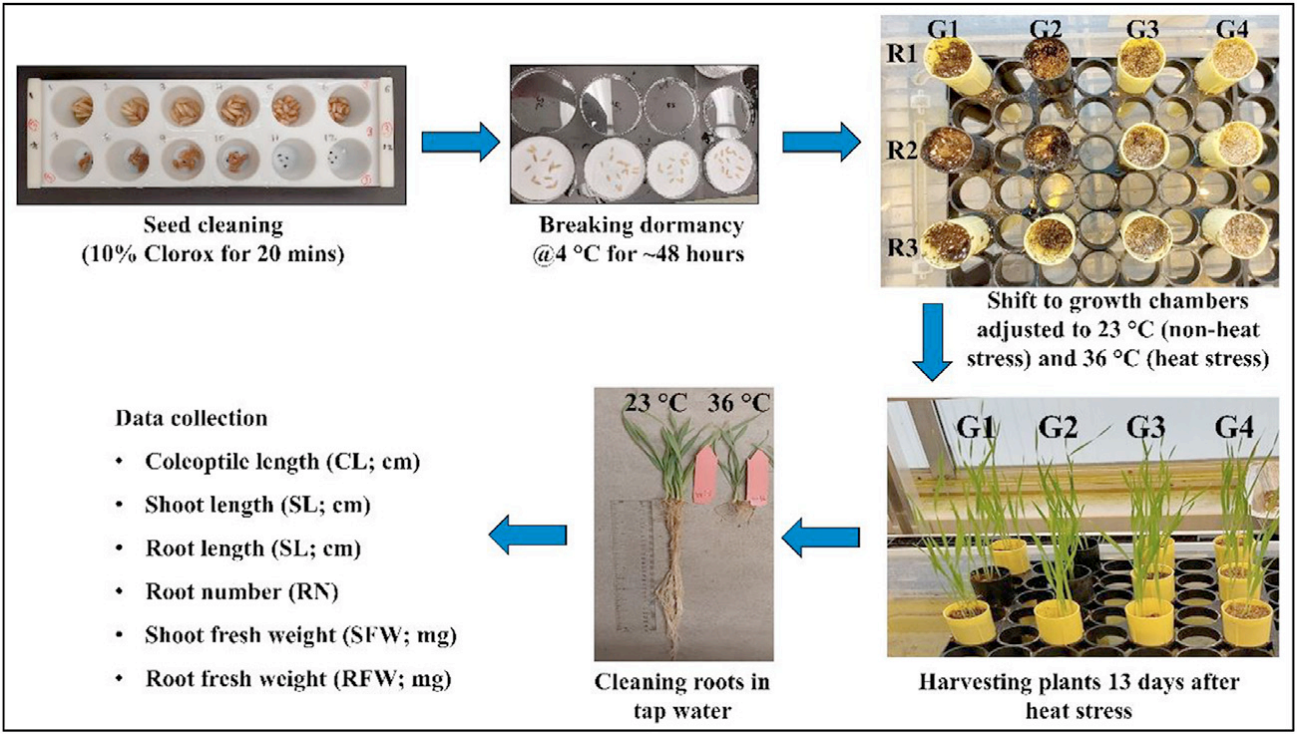
Protocol used for screening wheat germplasm lines under non-heat stress (23 °C) and heat stress (36 °C) treatments.

**Fig. 2:**
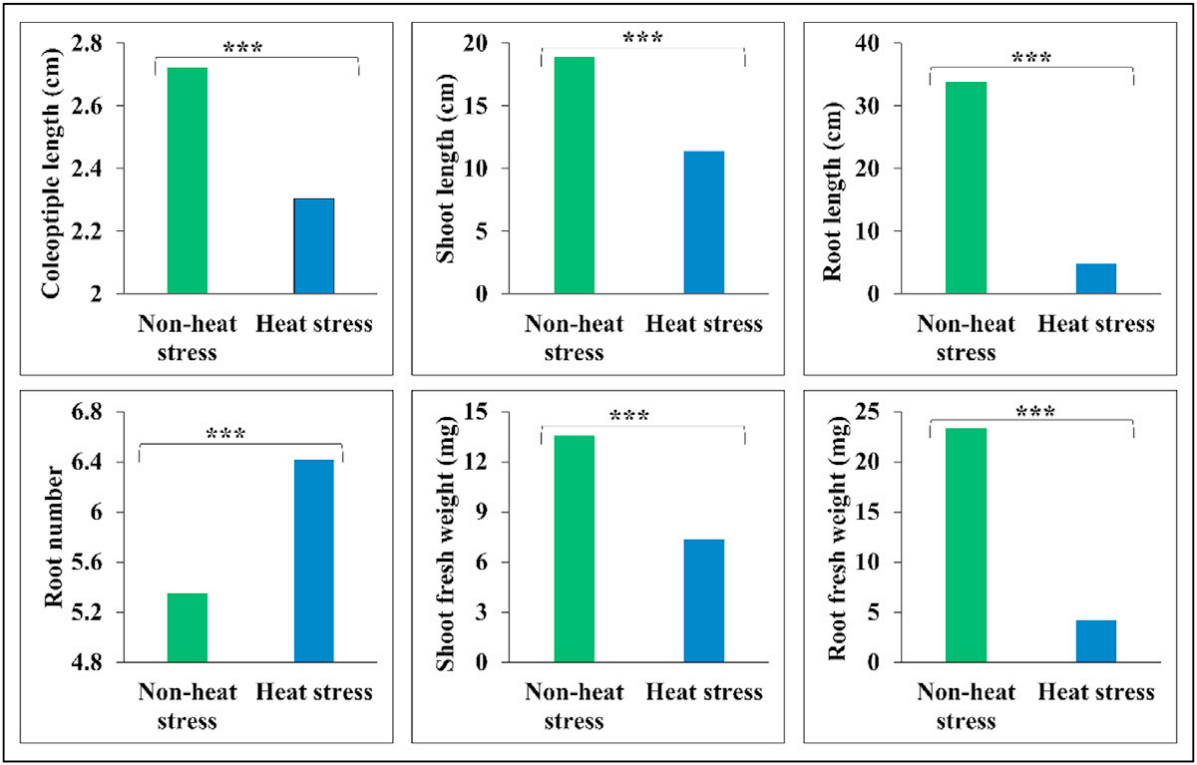
Comparing the performance of genotypes for various seedling traits under non-heat stress (23 °C; green bar) and heat stress (36 °C; blue bar) treatments. Level of significance: “*” at <0.05 %; “**” at <0.01 %; “***” at <0.001.

This data article describes the performance of genetically, phenotypically, and geographically diverse panel of 216 hexaploid spring wheat accessions evaluated under non-heat stress (23 °C) and heat stress (36 °C) conditions (Fig. 1). These germplasm lines were selected as the subset of exome-sequenced wheat panel, which consisted of diverse accessions of tetraploid and hexaploid wheat lines belonging to landraces and cultivars [7,14,15]. Screening was done under the controlled environment conditions using ‘Conviron’ growth chambers (Fig. 1). Plants were harvested 13 days after sowing and the data on various seedling traits such as coleoptile length (CL; cm), shoot length (SL; cm), root length (RL; cm), tiller number (TN), shoot fresh weight (SFW; mg), and root fresh weight (RFW; mg) were collected from non-heat stress (23 °C) and heat stress (36 °C) treatments. Genotypes showed huge variation for all seedling traits both under non-heat stress (23 °C) and heat stress (36 °C) treatments (Table 1). Heat stress significantly affected seedling performance by reducing CL (by 15.44 %), SL (by 39.85 %), RL (by 85.6 %), SFW (by 45.01 %), and RFW by (82.01 %) (Fig. 2). In contrast, RN was increased by 20 % under heat stress. These results suggests that the effect of heat stress was more in root traits (such as RL and RFW) than in shoots (such as SL and SFW). Furthermore, based on seedling performance, we identified six heat tolerant (i.e., PI 366,905, Kzyl Sark, Rang, Perico S, Bohr Gamh, and PI 620,689) and six heat sensitive (i.e., CItr 17,470, CItr 13,270, Coeruleum, Shashi, Hallany, and Currawa) genotypes. These genotypes can be utilized to develop bi-parental mapping populations, which helps in mapping and cloning heat tolerant genes. Moreover, heat tolerant genotypes identified in this study can serve as starting material for developing heat resilient wheat cultivars.

**Table 1:** Descriptive statistics for the seedling traits evaluated under non-heat stress (23 °C) and heat stress (36 °C) treatments. CV: coefficient of variance.

Statistic	Coleoptile length (CL; cm)	Shoot length (SL; cm)	Root length (RL; cm)	Root number (RN)	Shoot fresh weight (SFW; mg)	Root fresh weight (RFW; mg)
**Non-heat stress (23** °**C)**						
Grand mean	2.72	18.92	33.76	5.35	13.42	23.23
Range	1.87–3.68	10.8–31.92	17.33–48.5	3.43–7.67	1.14–23.4	4–46.82
CV ( %)	11.55	13.17	11.17	13.94	22.96	11.39
Heritability	0.77	0.83	0.89	0.66	0.82	0.96
Genotype	<0.05	<0.05	<0.05	<0.05	<0.05	<0.05
**Heat stress (36** °**C)**						
Grand mean	2.3	11.38	4.86	6.42	7.38	4.18
Range	1.1–3.42	5.17–20.67	1.6–10.6	3.67–9.67	1.33–17	0.4–16
CV ( %)	12.39	15.37	19.15	17.51	25.69	39.42
Heritability	0.82	0.83	0.93	0.71	0.88	0.85
Genotype	<0.05	<0.05	<0.05	<0.05	<0.05	<0.05
**Pooled analysis**						
Grand mean	2.51	15.15	19.31	5.88	10.4	13.7
Range	1.73–3.33	9.31–22.57	10.69–29.29	4.21–8.08	2.25–19.3	2.5–27.31
CV ( %)	11.95	14.24	14.22	16.22	25.01	16.11
Heritability	76.68	60.45	35.42	60.06	79.77	81.56
Genotype	<0.05	<0.05	<0.05	<0.05	<0.05	<0.05
Genotype x treatment	<0.05	<0.05	<0.05	<0.05	<0.05	<0.05
**Percent reduction**	15.44	39.85	85.6	−20	45.01	82.01

Genotypes showed significant variation (P-value < 0.05) for all seedling traits under non-heat stress and heat stress treatments as well as based on pooled analysis (Table 1). Phenotypic variation for seedling traits was more under heat stress than under non-heat stress as revealed by coefficient of variation (CV). CV ranges from 11.17 % (for RL) to 22.96 % (for SFW) under non-heat stress, whereas the CV ranges from 12.39 % (for CL) to 39.42 % (for RFW) under heat stress (Table 1). Broad sense heritability (h^2^_bs_) was higher under heat stress as compared to that under non-heat stress treatment for all the traits except for RFW. Broad sense heritability ranges from 66 % for RN to 96 % for RFW under non-heat stress, whereas it ranges from 71 % for RN to 93 % for RL under heat stress (Table 1). We also noticed a significant genotype-treatment (P-value < 0.05) interactions for all seedling traits, highlighting the effect of heat stress and non-heat stress on seedling performance.

Pearson’s correlation coefficient analysis revealed a significant (P-value < 0.05) positive correlation among different seedling traits under non-heat stress and heat stress treatments. Under non-heat stress most traits were positively correlated except for RL with CL, RN, and SFW, which were non-significantly correlated (Table 2). The strongest association was observed for SFW with SL and RFW (i.e., *r* = 0.7; P-values < 0.001) and the weakest association was observed between CL and RN (i.e., *r* = 0.22; P-values < 0.01). Under heat stress treatment all traits showed significant (P-value < 0.05) positive association (Table 3). The strongest association was observed between SFW and RFW (i.e., *r* = 0.67; P-values < 0.001), and the weakest association observed between RL and RN (i.e., *r* = 0.15; P-values < 0.05). Correlation coefficient results also showed a notable shift in the trait relationship across the treatments (Table 2, Table 3). For instance, RL was not significantly correlated with CL, RN, and SFW under non-heat stress treatment, whereas under heat stress, the RL showed a significant positive correlation with CL (*r* = 0.21; P-values < 0.01), RN (*r* = 0.15; P-values < 0.05), and RFW (*r* = 0.36; P-values < 0.001). Similarly, under non-heat stress SL and RL showed weak correlation (*r* = 0.18; P-values < 0.01), whereas the association among SL and RL was strong (*r* = 0.58; P-values < 0.001) under heat stress. Conversely, under non-heat stress CL and RFW showed strong correlation (*r* = 0.25; P-values < 0.01), whereas the association among CL and RFW was weak (*r* = 0.16; P-values < 0.05) under heat stress.

**Table 2:** Pearson's correlation coefficient analysis for seedling traits under non-heat stress (23 °C). Level of significance: “*” at <0.05 %; “**” at <0.01 %; “***” at <0.001.

Traits	Coleoptile length (CL; cm)	Shoot length (SL; cm)	Root length (RL; cm)	Root number (RN)	Shoot fresh weight (SFW; mg)	Root fresh weight (RFW; mg)
**Coleoptile length (CL; cm)**	1	–	–	–	–	–
**Shoot length (SL; cm)**	0.59***	1	–	–	–	–
**Root length (RL; cm)**	−0.01	0.18**	1	–	–	–
**Root number (RN)**	0.22**	0.39***	−0.09	1	–	–
**Shoot fresh weight (SFW; mg)**	0.42***	0.7***	0.1	0.42***	1	–
**Root fresh weight (RFW; mg)**	0.25**	0.58***	0.55***	0.35***	0.7***	1

**Table 3:** Pearson's correlation coefficient analysis for seedling traits under heat stress (36 °C). Level of significance: “*” at <0.05 %; “**” at <0.01 %; “***” at <0.001.

Traits	Coleoptile length (CL; cm)	Shoot length (SL; cm)	Root length (RL; cm)	Root number (RN)	Shoot fresh weight (SFW; mg)	Root fresh weight (RFW; mg)
**Coleoptile length (CL; cm)**	1	–	–	–	–	–
**Shoot length (SL; cm)**	0.56***	1	–	–	–	–
**Root length (RL; cm)**	0.21**	0.58***	1	–	–	–
**Root number (RN)**	0.21**	0.42***	0.15*	1	–	–
**Shoot fresh weight (SFW; mg)**	0.36***	0.64***	0.36***	0.38***	1	–
**Root fresh weight (RFW; mg)**	0.16*	0.41***	0.39***	0.4***	0.67***	1

## 4 Experimental Design, Materials and Methods

### 4.1 Experimental plan

The experiment was conducted at the at Edward T. Schafer Agricultural Research Center, Fargo, ND, USA (46.892593 N and −96.807467 W) using ‘Conviron’ growth chambers. The experiment was carried out using two-factor randomized complete block design (RCBD) with three replications. Two factors represent the two temperature regimes (such as 23 °C for non-heat stress treatment and 36 °C for heat stress treatment) used to screen the wheat genotypes. Clean, bold, and healthy seeds harvested from single plants from each genotype were surface sterilized to remove the microbial contamination (such as fungus and bacteria) [6,16]. For this purpose, we used 10 % Clorox for 20 min and 70 % ethanol for 5 min, followed by three-time washing (5 min each) using deionized water (diH_2_O). Surface sterilized seeds were kept in refrigerator (with 4 °C) for 48 h to overcome dormancy as well as to achieve uniform seed germination.

The experiment was conducted in 98 cone trainer trays (24″ L: 12″ W: 6.75″ H), each containing 98 plastic cones measuring 30 cm in length and 5 cm in diameter, with drainage holes at the bottom (Fig. 1). Cones were filled with wet autoclaved vermiculite (Gray coloured PVP horticultural medium sized vermiculite). Cold treated seeds from each genotype were sown in six cones. Three of the six cones representing three replications used for non-heat stress treatment (i.e., 23 °C), and the remaining three cones were used for heat stress treatment (i.e., 36 °C). Once sowing is completed, genotypes were randomized in two sets of trainer trays (one was used for non-heat stress treatment and another for heat stress treatment). Trainer trays were kept in the small flow trays (27″ L: 15.2″ W: 5″ H) to hold water and were immediately moved to growth chambers. One growth chamber used for non-heat stress treatment was adjusted to 23 °C temperature and the other one used for heat stress treatment was adjusted to 36 °C temperature, to ensure the heat stress from sowing itself. However, the other environmental factors such as humidity, light intensity, and light duration was kept same for both the chambers. Once seeds were germinated, both growth chambers were adjusted to a 16-hour photoperiod (i.e., 16 h of light and 8 h of dark). Precautions were taken to avoid water stress by providing sufficient amounts of cold water (23 °C) for non-heat stress treatment and warm water (36 °C) for heat stress treatment via flow trays. Moreover, containers were replaced with the fresh water after every 3rd day to avoid fungal growth.

### 4.2 Phenotypic data collection

Seedlings were allowed to grow up to 13 days after sowing. Plants were harvested on 13th day and the roots were cleaned using running tap water. Data was collected on various seedling traits such as coleoptile length (CL; cm), shoot length (SL; cm), root length (RL; cm), root number (RN), shoot fresh weight (SFW; mg), and root fresh weight (RFW; mg). CL was measured from scutellar node to the tip of coleoptile. SL was measured from scutellar node to the tip of topmost leaf, whereas the RL was measured from scutellar node to the tip of primary root. RN was determined by counting the main roots emerging from the radicle for each plant. Roots and shoots were separated by cutting plants at the scutellar node and the data on SFW and RFW were measured.

### 4.3 Statistical analysis for seedling traits

Seedling data collected under non-heat stress (23 °C) and heat stress (36 °C) treatments were subjected to mixed linear model (MLM) analysis using META-R software [17]. The META-R uses LME4, an R-package to calculate the genotypic best linear unbiased estimates (BLUEs) for individual treatments.

BLUE values were estimated by using the following model:

$$Y_{\text{in}}=\mu +G_{1}+R_{n}+e_{\text{in}}$$

Where, Y_in_ is trait of interest, µ is the grand mean, G_i_ is the effect of i^th^ genotype, R_n_ is the effect of n^th^ replication, e_in_ is the error associated with i^th^ genotype and n^th^ replication.

Coefficient of variance (CV) was calculated by using the formula:

$\text{Coefficient}\;\text{of}\;\text{variance}\;\left(\text{CV};\%\right)=\frac{\sigma }{\mu }\;\times \;100$

Where, σ is the standard deviation, µ is the mean.

Broad-sense heritability (h^2^_bs_) was calculated by using the following formula:

$${h_{bs}}^{2}=\frac{\sigma^{2}g}{\sigma^{2}g+\sigma^{2}e/R}$$

Where, σ^2^_g_ is genetic variance, σ^2^_e_ is error variance, R is number of replications

Pearson’s correlation coefficient analysis among the seedling traits were assessed based on BLUE values using “Corrplot” package built in the RStudio [18].

## Limitations

This dataset includes the impact of heat stress on various seedling traits only. However, heat stress affects wheat at various developmental stages including flowering and grain filling stages. Future studies must focus on evaluating the impact of heat stress during later development stages also.

## Ethics Statement

Authors have read and follow the ethical requirements for publication in Data in Brief and confirming that the current work does not involve human subjects, animal experiments, or any data collected from social media platforms.

## Credit author statement

**Santosh Gudi:** Conceptualization, Data analysis, Visualization, Writing- original draft preparation, Writing- review and editing. **Jatinder Singh:** Data analysis. **Harsimardeep S Gill:** Data analysis. **Sunish K Sehgal:** Writing- review and editing. **Justin D Faris:** Writing: review and editing**. Upinder Gill:** Writing- review and editing. and **Rajeev Gupta:** Conceptualization, Writing- review and editing, Supervision. All authors have read and agreed to this version of the manuscript.

## Data Availability

Mendeley DataDataset for seedling traits evaluated under non-heat stress (23 °C) and heat stress (36 °C) conditions in hexaploid spring wheat panel (Original data).
